# Disruption of Early or Late Epochs of Auditory Cortical Activity Impairs Speech Discrimination in Mice

**DOI:** 10.3389/fnins.2019.01394

**Published:** 2020-01-10

**Authors:** Conor O’Sullivan, Aldis P. Weible, Michael Wehr

**Affiliations:** ^1^Institute of Neuroscience, University of Oregon, Eugene, OR, United States; ^2^Department of Biology, University of Oregon, Eugene, OR, United States; ^3^Department of Psychology, University of Oregon, Eugene, OR, United States

**Keywords:** consonant, speech – brain, speech perception, discrimination, auditory cortex, neural coding, spike time coding, optogenetic

## Abstract

Speech evokes robust activity in auditory cortex, which contains information over a wide range of spatial and temporal scales. It remains unclear which components of these neural representations are causally involved in the perception and processing of speech sounds. Here we compared the relative importance of early and late speech-evoked activity for consonant discrimination. We trained mice to discriminate the initial consonants in spoken words, and then tested the effect of optogenetically suppressing different temporal windows of speech-evoked activity in auditory cortex. We found that both early and late suppression disrupted performance equivalently. These results suggest that mice are impaired at recognizing either type of disrupted representation because it differs from those learned in training.

## Introduction

Speech activates broad spatiotemporal patterns of activity throughout the mammalian auditory system (Johnson et al., 2005; Liebenthal et al., 2005; Kilgard and Engineer, 2015). The distinct neural representations evoked by different speech sounds are thought to underlie our ability to distinguish between them (Engineer et al., 2008; Centanni et al., 2013; Kilgard and Engineer, 2015). However, it is still unclear which details of these spatiotemporal activity patterns matter, and how neural processing of them leads ultimately to perceptual discrimination. Early in the auditory pathway, speech-evoked neural activity appears to encode the acoustic structure of speech sounds, whereas in higher order auditory areas, it appears to encode progressively more categorical aspects of speech (Chang et al., 2010; Flinker et al., 2011; Tsunada et al., 2011, 2016; Pasley et al., 2012). Partway along this hierarchical transformation, core auditory cortex gives rise to the dorsal and ventral processing streams, and contains both temporal and rate coded representations of speech (Rauschecker and Scott, 2009; Perez et al., 2013).

It is clear from lesion studies that auditory cortex is necessary for the discrimination of complex sounds, including speech, but not for simpler auditory tasks such as frequency discrimination (Cranford et al., 1976; Ohl et al., 1999; Floody et al., 2010; Porter et al., 2011). Converging evidence from electrophysiology, behavior, and lesion studies has implicated precise spike timing during the initial onset response in auditory cortex as being especially important for the discrimination of consonants. In rats trained to discriminate consonants, their discrimination performance is well correlated with the distinctness of spatiotemporal activity patterns in auditory cortex (Engineer et al., 2008; Centanni et al., 2013). Precise spike timing is critical for this relationship, because the removal of spike timing information by binning responses disrupts both neural discriminability and its correlation with behavioral discrimination (Schnupp et al., 2006; Engineer et al., 2008; Centanni et al., 2013). The initial 40 ms of the onset response in auditory cortex appears to be particularly informative. For neural decoding of speech sounds, the initial 40 ms of the response contains the most information for neural discrimination of consonants (Engineer et al., 2008; Perez et al., 2013; Centanni et al., 2014). In addition, auditory cortex lesions cause much greater impairment of the discrimination of speech sounds that are truncated to the initial 40 ms than for full syllables (Porter et al., 2011).

The picture that emerges from these findings is that not all of the neuronal spiking in a speech-evoked spatiotemporal pattern of cortical activity is equipotent for discrimination of consonants. Rather, early activity appears to contain more discriminative information than later activity. However, the hypothesis that early cortical activity matters more for discrimination of speech sounds than late activity has not yet been rigorously tested. Speech-evoked responses of cortical neurons show higher correlation with behavioral discriminability during the initial 40 ms of activity (Engineer et al., 2008), but this correlation does not demonstrate a causal relationship with behavioral discrimination. The fact that cortical lesions cause greater impairment for discrimination of truncated speech sounds demonstrates a causal role for auditory cortex (Porter et al., 2011), but manipulating the stimulus is not the same thing as manipulating the neural representation itself. Here we set out to test this hypothesis by taking advantage of the temporal precision of optogenetics to manipulate cortical activity during the discrimination of speech sounds. Mice discriminated the words “dad” and “sad,” pitch-shifted up into the mouse hearing range. These sounds are most different in the early 0–140 ms time window (initial consonant “d” or “s”) but are much more similar in the late 140–280 ms time window (during the rhyming vowel sound). We predicted that suppressing early speech-evoked activity in auditory cortex would cause a greater impairment than suppressing late activity. We found that full suppression over the entire duration of the stimulus partially but significantly impaired discrimination, confirming previous results from lesion studies. Surprisingly, we found that suppression during the early or late temporal windows was equally disruptive, each causing as much impairment as full suppression. We conclude that for mice trained to discriminate speech sounds, disruption of any temporal component of the representation impairs discrimination. Our interpretation is that regardless of which spatiotemporal aspects of a representation may be most informative, any type of disrupted representation differs from those learned in training, thereby impairing performance.

## Materials and Methods

All procedures were performed in accordance with the National Institutes of Health guidelines, as approved by the University of Oregon Institutional Animal Care and Use Committee.

### Mice

In this study, we used a total of nine mice for optogenetic suppression of auditory cortex during performance of a phoneme discrimination task. The mice were offspring from a cross of homozygous Pvalb-IRES-Cre (“PV,” JAX No. 008069; The Jackson Laboratory) and homozygous CAG-ChR2-eYFP (“ChR2,” JAX No. 012569; The Jackson Laboratory) lines, which are on a C57Bl6/J background. In these mice (“PV-ChR2”), Channelrhodopsin2 (ChR2) was expressed in parvalbumin-expressing (PV+) interneurons (Moore and Wehr, 2013). We used an additional 2 PV-ChR2 mice (not used for behavior) for electrophysiological validation of optogenetic suppression. After training (see below), at the beginning of optogenetic suppression, mice were 5.5 months of age (median; range: 4.0–7.2 months). On the last day of data acquisition, mice were 7.7 months of age (range: 5.8–14.9 months). At these ages, C57BL/6J mice have likely developed significant age-related high-frequency hearing loss (Ison et al., 2007). Accordingly, we presented stimuli at 70–80 dB SPL (see below), which should be well above threshold even in the presence of age-related hearing loss.

### Surgical Procedures

To deliver laser illumination to auditory cortex while mice performed the task, we surgically implanted optical fibers bilaterally before the beginning of training. We administered dexamethasone (0.1 mg/kg) and atropine (0.03 mg/kg) pre-surgically to reduce inflammation and respiratory irregularities. Surgical anesthesia was maintained with isoflurane (1.25–2.0%). We implanted 200 μm optic fibers in each hemisphere at AP ∼2.3 mm (relative to bregma), ML 4.4 mm, and depth 0.5 mm below the dura (just dorsal to primary auditory cortex). The implants were painted with black acrylic paint to minimize light leakage. For electrophysiological verification of optogenetic suppression, we implanted two mice (not used in behavioral experiments) with a unilateral optrode array, consisting of eight tetrodes and a 200 μm fiber terminating 1 mm above the recording sites. The eight tetrodes passed through two 28-gauge stainless steel hypodermic tubes, with four tetrodes per tube. The optic fiber was fixed in position immediately adjacent to, and between, these tubes. Tetrodes were made of 18 μm (25 μm coated) tungsten wire (California Fine Wire). The entire array was mounted on a custom microdrive. The optrode array was inserted vertically through a small craniotomy (2 mm × 1 mm) dorsal to auditory cortex, and cemented into place. We administered ketoprofen (4.0 mg/kg) post-operatively to minimize discomfort. Mice were housed individually following the surgery and were allowed 7 days of post-operative recovery.

### Histology

Brains of mice used for electrophysiological validation were sectioned (100 μm) in the coronal plane to verify the position of single neuron recording sites. Only data corresponding to tracks located within auditory cortex were included. Following behavior experiments, the brains of six out of the nine mice were sectioned to confirm the location of implanted fibers in auditory cortex.

### Behavioral Apparatus

**FIGURE 1:**
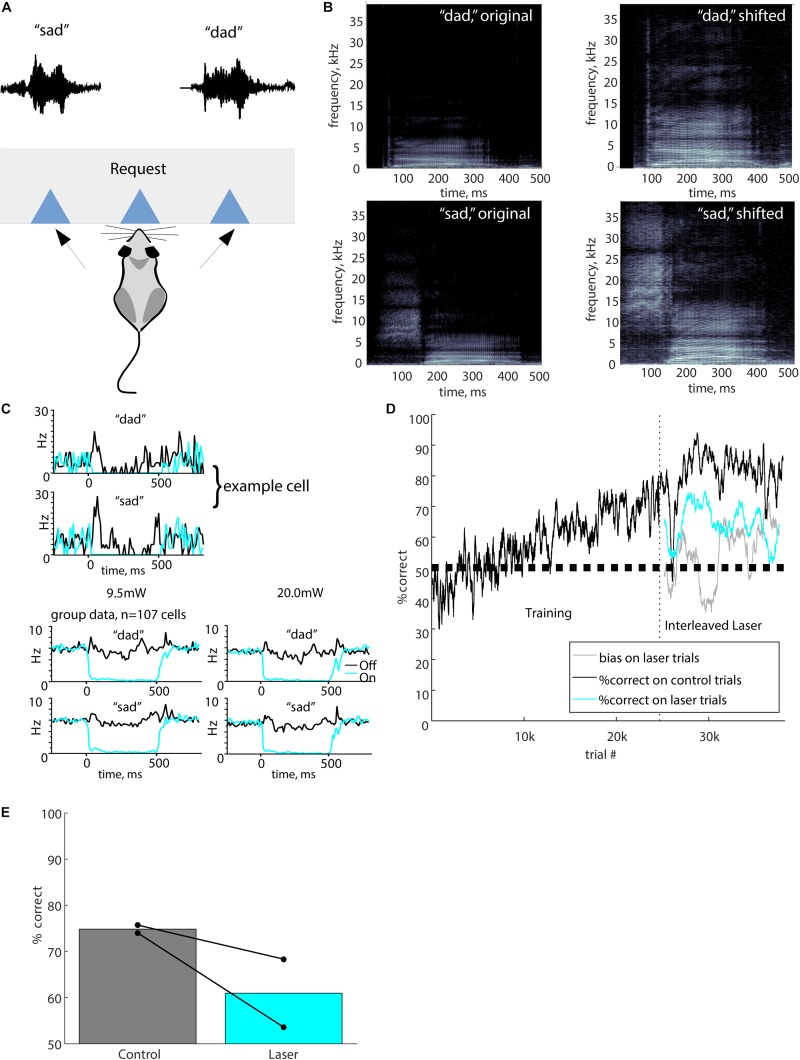
Full stimulus suppression. **(A)** The port mapping for this task involved a central lick to initiate a trial, which triggered playback of either “sad” or “dad.” The waveforms for the “sad” and “dad” stimuli shown above the corresponding response port. A reward for a correct response was delivered at the response port. **(B)** Spectrograms of the original human voice recordings of “dad” and “sad” are shown alongside the pitch-shifted (up one octave) stimuli used for behavioral testing. In all plots, grayscale represents the power spectral density (dB/Hz), with black representing -110 dB/Hz and white representing -30 dB/Hz. **(C)** Optogenetic suppression produced nearly complete silencing of neurons in auditory cortex. The upper panels show responses of an example cell to the speech stimuli used in the task (black), and complete suppression of these responses by laser illumination (cyan, 500 ms at 9.5 mW). Both stimulus and laser onset occurred at time 0. Bottom panels show group data averaged across 107 cells in two mice, at both 9.5 and 20 mW laser powers. Cells were recorded with chronic tetrodes in two awake mice that were not used for behavior. **(D)** Performance of a typical mouse from initial training stages through laser suppression. Vertical dashed line indicates the onset of laser trials. Performance is represented as percent correct averaged across both stimuli in a 200-trial sliding window. Black, performance on control trials; cyan, performance on laser trials; gray, bias on laser trials. **(E)** Performance in overall percent correct for each mouse for 10,000 total trials is represented by connected dots representing control trials and suppression trials. Bars show mean performance for each trial type across both mice.

Mice performed the task in sound-attenuating behavioral chambers. Within the chamber, mice were placed in a plastic arena, one wall of which contained three combination ports for lick-sensing and water delivery (Figure 1A). Each port had an IR beam-break sensor, at which mice responded by licking, and a tube to deliver water rewards for correct responses. Sound stimuli were controlled by a computer running custom behavioral software modified from Meier et al. (2011), and delivered through two free-field JBL Duet speakers (high-frequency rolloff: 34 kHz) placed outside the arena facing the ports. Since laser illumination was delivered with blue light that could potentially be visible to the mouse, we used a color-matched blue strobe light (full-field illumination at approximately 10 Hz) to mask laser stimulation. Mice were trained for an hour each day for 5–7 days a week, corresponding to 300–500 trials and 1–2 g of water reward per day. Mice were water-restricted, typically receiving water only through performance of the task, but were supplemented as necessary to remain above 80% of pre-training body mass.

### Stimuli

We recorded the words “sad” and “dad,” spoken by a female native speaker of US English, using a Bruel and Kjaer 4939 microphone. We digitized the signal at 176 kHz and then decimated down to 44.1 kHz. Because much of the frequency content of human speech is below the mouse hearing range (which is approximately 1–80 kHz), we pitch-shifted the speech upward by one octave using a frame-based algorithm with Fourier transforms of window length 2048, analysis length 512, and synthesis length 1024 samples (Saunders and Wehr, 2019). The resulting pitch-shifted stimuli had preserved temporal structure and a sample rate of 88.2 kHz (Figure 1B). Stimuli were delivered at approximately 70–80 dB SPL (RMS). Because the initial consonants /s/ and /d/ were of different durations, we added 50 ms of silence to the beginning of the “dad” stimulus so that the consonant-to-vowel transition occurred at approximately 140 ms for both stimuli, and both stimuli had a total duration of 500 ms.

### Task Structure

Prior to any behavioral training, the mice underwent surgical implantation of optical fibers (Step 1, see Table 1). After recovery, the mice were familiarized with the operation of the ports in the absence of sound stimuli using a simple lick-for-water task (Step 2, “Free drinks”). Next, they advanced to the first stage of the main task (Step 3). In the main task, mice requested trials by licking the center port, which triggered stimulus delivery. Mice responded by licking at the left port (for “sad”) or the right port (for “dad”). Correct responses triggered an 80 μl water reward followed by a 1 s delay before the next trial could be requested, whereas incorrect responses gave no water and provided an additional 1 s penalty timeout before the next trial. To increase the number of trials performed, some mice had their water rewards reduced to 60 or 40 μl. During an initial shaping stage of the main task (Step 3), mice received water rewards at the center port for requesting trials (as well as for correct answers at the side ports) until reaching a rate of seven completed trials in 30 s. Once the mice achieved this rate of trials, the rewards for center-poke trial requests were removed, forming the next stage (Step 4) of the task structure. After 400 trials in this condition, the penalty timeout for incorrect responses was increased to 3 s (Step 5). In Steps 3–5, we included “correction trials” to reduce the development of response bias to one side or the other. After an incorrect response, there was a 50% chance that a mouse would go into a correction trial sequence, in which the same stimulus was repeated until the mouse responded correctly. Correction trials provide contextual information that could conceivably allow a task strategy that did not depend solely on stimulus discrimination, so we disabled correction trials during the final optogenetic suppression stage (Step 6). When mice were performing at 70% or higher on Step 5 for approximately 5 days, they were run for at least 2 days with fibers attached but without light delivery, to allow the mice to become accustomed to the fibers. Then mice advanced to the final stage (Step 6) for optogenetic suppression experiments.

**TABLE 1:** Training steps.

**Step**	**Description**	**Advancement criteria**
1. Surgery	Fiber implantation	1 day of water restriction post-recovery
2. Free drinks	Ports give water, no stimulus	Trial rate
3. Request rewards	Rewards for center port trial requests and correct responses	Trial rate
4. Only correct rewards	Request rewards disabled	400 trials completed
5. Long penalty	Increased timeout for incorrect responses	Performance > ∼70%
6. Optogenetic suppression	Laser on for 10% of trials	N/A

### Optogenetic Suppression

**FIGURE 2:**
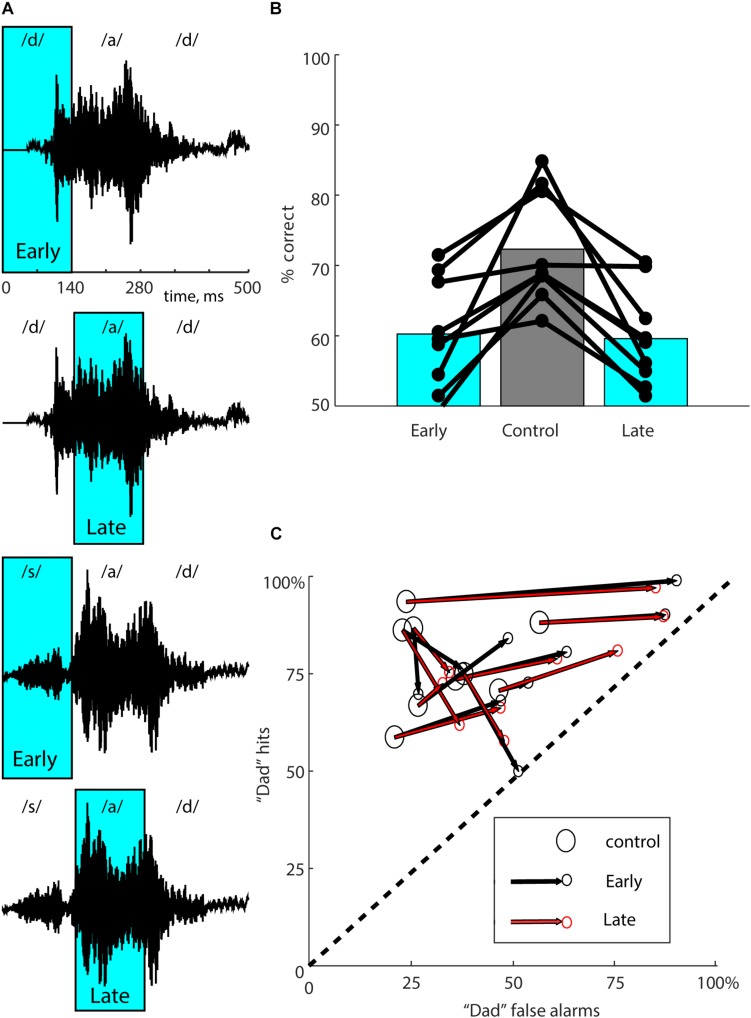
Multiple suppression windows. **(A)** The waveforms for both stimuli (“dad” in the two upper panels, “sad” in the two lower panels) are shown with the “Early” and “Late” laser suppression windows overlaid in cyan. Approximate locations of the phonemes within each stimulus are indicated above each waveform. Early suppression coincides with the initial consonant (/d/ or /s/), and Late suppression coincides with the vowel (/a/). **(B)** Performance in overall percent correct for each mouse for 10,000 total trials is represented by connected dots representing each of the three types of trials. Bars represent mean performance for each trial type across all nine mice. Mice showed similar effects from either Early or Late suppression. **(C)** To partition suppression effects into accuracy and bias, performance for each mouse (same data as in **B**) was separated into “dad hit rate” (percent correct on “dad” trials) and “dad false alarm rate” (100 - percent correct on “sad” trials). Each mouse is represented with a large open circle corresponding to control trials, connected to small circles corresponding to Early (black) and Late (red) suppression conditions. The dashed line represents chance performance (50% correct). The lower left-hand corner (0,0) represents 100% “sad” responses (i.e., total bias toward “sad”). The upper right-hand corner (100, 100) represents 100% “dad” responses (i.e., total bias toward “dad”). Optogenetic suppression reduced accuracy for all mice, shifting performance toward the dashed line. The amount of response bias on suppression trials ranged from almost none (a shift perpendicular to the dashed line) to a strong bias toward “dad” (a shift toward the upper right-hand corner). If the effect of the laser was exactly the same for both suppression windows, the two small red and black circles would coincide. The effects of Early and Late suppression windows were very similar.

To suppress auditory cortex, we delivered 445 nm wavelength laser pulses to auditory cortex through chronically implanted bilateral optical fibers. We used two laser powers: a standard total power of 20 mW (corresponding to 630 mW/mm^2^ at the fiber tip) and additional testing with a total power of 9.5 mW (300 mW/mm^2^). In a previous study using identical fiber implantation and lasers, we electrophysiologically characterized the spatial extent of cortical suppression, which was 1750 μm at a power of 9.5 mW (Weible et al., 2014). We have not characterized the spatial extent of suppression at 20 mW, but based on our previous measurements at 9.5 mW and a model of light transmission in mammalian brain tissue^1^, we estimate that the spatial extent of suppression at 20 mW is 2100 μm, an extent that includes all tonotopic fields of auditory cortex, throughout the cortical depth, but does not include thalamic, collicular, or other subcortical auditory structures. Laser illumination trials were randomly interleaved on 10% of trials. We used three temporal profiles for illumination. For “Full” suppression trials, illumination was delivered during the entire 500 ms from sound onset to sound termination. For “Early” suppression trials, illumination was delivered from 0 to140 ms following stimulus onset, whereas for “Late” trials, illumination was delivered from 140 to 280 ms following stimulus onset (Figure 2A). Because the consonant-to-vowel transition occurred at ∼140 ms, Early suppression coincided with the initial consonants (/d/ or /s/), and Late suppression coincided with the following vowel. We did not include a condition that specifically targeted the trailing consonant /d/. Initially, all laser trials were rewarded randomly in order to avoid the possibility that mice might be able to learn new associations between laser + stimulus and rewards (if laser + stimulus combinations were correctly rewarded according to the stimulus identity). In follow-up experiments, we explicitly examined whether mice could learn new associations by correctly rewarding laser trials.

As an additional control experiment, we tested the possibility that visible cues produced by the laser could influence performance through learning. In this case, instead of connecting the optical fibers to the implanted optical ferrules, we placed them on the wall of the behavioral chamber, where the light was clearly visible to the mouse, but not reaching the brain. We tested this condition with both randomly rewarded laser trials and correctly rewarded laser trials to examine the ability of mice to learn visible laser-cue associations under both reward conditions.

### Statistical Analysis

**FIGURE 3:**
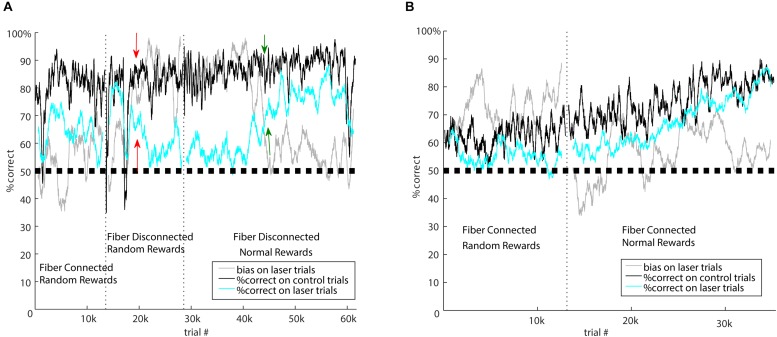
Laser-related learning. **(A)** Mouse 5982 went through three different forms of the task: first with standard laser suppression (random rewards), then with fibers disconnected and random rewards, then finally with fibers disconnected and normally rewarded laser trials. Vertical dotted lines indicate the transitions between these segments. Performance is represented as percent correct averaged across both stimuli in a 100-trial sliding window. Black, performance on control trials; cyan, performance on laser trials; gray, bias on laser trials. Performance on control trials remained consistent over time, but performance and bias on laser trials were sensitive to the specific task conditions. During standard suppression, there was a ∼15% impairment in performance on laser trials, but when the fibers were disconnected from the brain, there was an initial period during which laser had no effect on performance. In this condition, the salient visual laser cue indicates random rewards. After several thousand trials, indicated by the red arrows, this mouse developed a strong bias. One interpretation of this is that the mouse learned that its responses were irrelevant on laser trials, and adopted a bias strategy to minimize effort on those trials. In the final segment, we rewarded laser trials normally, which reinforced responses on laser trials. Over time, performance on laser trials became indistinguishable from control, and the bias diminished; green arrows indicate the approximate time of the major shift in performance. **(B)** After initial testing showed a moderate effect of the standard laser suppression, mouse 5916 was tested with normally rewarded laser trials during optogenetic suppression of auditory cortex. The onset of normal rewards on laser trials is indicated by the vertical dotted line. After this, the impairment on laser trials persisted for several thousand trials, but then diminished. By about 15k trials after the onset of normal rewards, performance on control and laser trials was indistinguishable. This suggests that this mouse was eventually able to learn to respond correctly even when auditory cortex was suppressed.

We calculated performance separately for responses on laser and control trials. Because not all performance data were normally distributed (Lilliefors test), we used non-parametric statistics throughout. We compared accuracy (in percent correct) for individual mice using Fisher’s exact test on the contingency tables created by the two stimuli and two possible responses, using the odds ratio as a measure of effect size. We tested for group effects using the two-tailed Wilcoxon signed-rank test. Because we found a range of long-term learning effects (as described in section “Laser-Related Learning” and in Figure 3), we used only the first 10,000 total trials after the start of laser stimulation (Step 6) unless otherwise noted. To examine changes over time, we calculated performance using a 200-trial sliding window.

To determine the rightward or leftward response bias of each mouse, we calculated the difference between the proportion of rightward responses and rightward stimuli (i.e., stimuli for which the correct response is rightward) in a sliding 200-trial window. A difference of zero indicates no bias, such that responses are proportionate to the stimuli presented. For display, we added 50% to the bias values, such that 50% indicates no bias, values < 50% indicate leftward bias, and values > 50% indicate rightward bias.

For receiver-operator-characteristic (ROC) analysis (Green and Swets, 1966), we evaluated performance separately for each stimulus and then compared between laser and control trials. To show stimulus-related effects, each mouse’s performance was separated into “hit rate” (percent correct on “dad” trials) and “false alarm rate” (100 - percent correct on “sad” trials). This arbitrary assignment to hits and false alarms allows ROC analysis of laser effects on both accuracy and response bias. For each mouse, we plotted hits against false alarms separately for laser and control conditions (Figure 2C). The distance between these points indicates the magnitude of the laser effect on accuracy, and the direction between them indicates the degree of induced bias.

### Single Neuron Recording and Analysis

Tetrode data were acquired with 32-channel RHD2000 hardware (Intan Technologies) and Open Ephys software (Siegle et al., 2017). A minimum threshold of 60 μV was set for collection of spiking activity. Activity of individual neurons was isolated offline using MClust (Redish, 2008). Measures of peak and trough waveform voltage, energy, and principal components analysis were used as waveform separation parameters in two-dimensional (2-D) cluster space. Cells were accepted for analysis only if they had a cluster boundary completely separate from adjacent cluster boundaries, and completely above threshold, on at least one 2-D view. Cluster boundaries were then applied across sessions to track single cell responses across different stimulus contingencies.

We recorded neuronal responses to the pitch-shifted “sad” and “dad” stimuli used for behavior (500 ms duration, 500 ms inter-trial interval, 50 repetitions) with or without optogenetic suppression on interleaved trials. We used the same laser power as in the behavior experiments (20 and 9.5 mW, corresponding to an irradiance of 630 and 300 mW/mm^2^ as measured at the tip of the 200 μm diameter fiber). All data were collected as mice freely explored a cylindrical plastic container (height 16 cm, diameter 16 cm) inside a double-walled sound-attenuating chamber. Sounds were delivered from a free-field speaker directly above the cylinder. The speaker was calibrated to within ± 1 dB using a Brüel and Kjaer 4939 1/4-inch microphone positioned within the cylinder approximately at head height. Following each recording session, the tetrode array was lowered ∼80 μm and allowed to settle for a minimum of 3 h before initiating another session to ensure that responses collected during each session reflected the activity of a unique population of cells. Recordings from putative PV cells, as identified by significant firing rate increases during laser pulses in silence compared with an equivalent period of silence with laser off (paired *t*-test), were excluded from group analyses.

## Results

### Training

Out of an initial 15 mice cohort implanted and trained on the task, nine successfully learned the task, reaching and maintaining 70–80% performance. From the start of training, it typically took 25–30k trials before mice advanced to optogenetic suppression experiments, corresponding to approximately 2–3 months.

### Electrophysiology

To verify that our optogenetic method effectively suppressed cortical activity, we recorded from auditory cortical neurons in two separate mice (not used for behavior) using a tetrode array, attached to an optical fiber implanted in the same location as the mice used for behavior. We recorded from 116 neurons. We excluded nine PV cells, which were unambiguously identified by robust responses to illumination (Moore and Wehr, 2013). Figure 1C shows a typical example of responses to the “dad” and “sad” speech stimuli, with complete optogenetic suppression by 300 mW/mm^2^ (9.5 mW) laser illumination. Across the population, suppression was nearly complete for both 300 and 630 mW/mm^2^ laser illumination (Figure 1C, bottom). Because neurons varied in the timing of their responses to the speech stimuli, the brisk transient responses seen in individual cells (Figure 1C, top) are not as evident in the population-averaged response (Figure 1C, bottom). However, the population average shows the effectiveness of optogenetic suppression. Across the population of 107 non-PV cells, suppression reduced the mean firing rate during phoneme presentation from 5.8 ± 5.5 to 0.6 ± 1.6 Hz (mean ± SD, *t* = 18.8, *p* < 0.0001, paired *t*-test) at an intensity of 300 mW/mm^2^, and from 5.1 ± 5.4 to 0.4 ± 1.1 Hz (mean ± SD, *t* = 17.4, *p* < 0.0001) at an intensity of 630 mW/mm^2^. Responses were significantly suppressed by illumination in 100/107 (93%) non-PV neurons.

### Full Stimulus Suppression

We first used “Full” illumination to verify that suppression of auditory cortex during the entire stimulus impaired the discrimination of speech sounds. We tested two mice, with an example training course shown in Figure 1D, using Full stimulus suppression in which 20 mW illumination was delivered during the entire 500 ms from sound onset to sound termination. Laser trials were randomly interleaved on 10% of trials. Performance was significantly impaired on laser trials compared to control trials (Figure 1E, 74.8 ± 2% on control trials, 60 ± 7% on laser trials). Both mice showed significant individual effects (mouse 5623: *p* < 0.0001, mouse 5625: *p* = 0.0002, Fisher’s exact test). Performance on laser trials (60%) remained significantly above chance (60 ± 7 vs. 50%, *p* = 10^–8^, Wilcoxon signed-rank), indicating that suppression partially but significantly impaired discrimination. Since there was a significant deficit when auditory cortex was suppressed, these results show that auditory cortex activity is required for normal discrimination of these speech sounds. Although our sample size was small, this result serves as a confirmation and agrees with previous lesion studies showing the necessity of auditory cortex for speech discrimination.

### Early and Late Suppression Windows

We used two speech stimuli that rhyme (“dad” and “sad”). That is, the initial consonant differed (/d/ vs. /s/), but the vowel and final consonant were the same (“ad”). We therefore wondered whether neural activity evoked by the initial consonant might be more informative for discrimination than neural activity evoked by the vowel and final consonant. Even though the acoustic fine structure of the trailing “ad” is slightly different in the two speech tokens, we reasoned that they were much more similar than the initial consonants, and therefore that the neural activity they evoke might provide less discriminative information. Initial onset-evoked cortical activity has also been shown to be most informative for consonant discrimination (Engineer et al., 2008; Centanni et al., 2013). To test this possibility, we took advantage of the temporal precision of optogenetics to suppress auditory cortical activity just during the initial 0–140 ms of the stimulus (containing /d/ or /s/, which we refer to as “Early” suppression, see Figure 2A), or instead during the trailing 140–280 ms of the stimulus (containing “ad,” which we refer to as “Late” suppression, see Figure 2A). Randomly interleaved Early and Late suppression trials each made up 10% of the total, with Control trials (no illumination) as the remaining 80%. We predicted that Early suppression would have a stronger effect on behavioral performance than Late suppression. We tested a group of nine mice with 20 mW illumination, including the two used in full suppression experiments. Surprisingly, we instead found that both Early and Late suppression produced similar deficits in performance (Figure 2B; Early: 60.2 ± 7.9%, Late: 59.6 ± 6.9%, Control: 72.3 ± 4.6%, *n* = 9 mice). Both Early and Late suppression were significantly different from Control (Early vs. Control: *p* = 0.0117, Late vs. Control: *p* = 0.0117, Wilcoxon signed-rank, corrected for multiple comparisons), whereas Early and Late suppression were not different from each other (*p* = 0.82). Performance on laser trials remained significantly above chance (Early: 60.2 vs. 50%, *p* = 0.004, Late: 59.6 vs. 50%, *p* = 0.002, Wilcoxon signed-rank). We conclude that either epoch of suppression was sufficient to disrupt performance.

To examine these effects in more detail, we quantified the effects of both Early and Late suppression using ROC analysis (Figure 2C), which allowed us to partition the effects on performance into effects on accuracy and bias. Out of the nine mice, seven showed significant effects of the laser in both conditions, one mouse had a significant effect only for Late suppression, and one did not show significant effects in either condition (Fisher’s exact test, *p* < 0.05). Most mice exhibited a side bias on laser trials, indicated by shifts in performance toward the upper-right corner in Figure 2C. Other mice showed almost no bias, with performance shifted toward the lower-right corner, corresponding to similar deficits for both stimuli. When mice did show a response bias it was predominantly toward “dad,” but it is not clear from these data whether this is somehow related to the acoustic structure of the stimuli, specific effects of optogenetic suppression, or just coincidence. Comparison of the effects of Early and Late suppression for each mouse showed that the directions and magnitudes were largely similar for both suppression windows. In other words, there was no difference between the locations of the small red and black circles for each mouse in Figure 2C [*p* = 0.76, multivariate paired Hotelling’s *T*^2^ = 4.02, *F*(2,7) = 1.76]. We conclude that the effects of the two suppression windows on performance were nearly equivalent in terms of both accuracy and bias.

To be able to directly compare with previous work, in which we electrophysiologically characterized the spatial extent of suppression at a laser power of 9.5 mW (Weible et al., 2014), we tested an additional mouse with 9.5 mW illumination. Discrimination in this mouse was partially but significantly impaired by 9.5 mW illumination (75.7% on control trials, 65.8% on laser trials averaged across both Early and Late, *p* < 0.0001, one-tailed Fisher’s exact test, first 10,000 trials), confirming that suppression with a known spatial extent limited to auditory cortex impaired performance.

### Laser-Related Learning

#### Disconnected Fibers

Because laser illumination of the brain is potentially visible to the mice during task performance, we performed three control experiments on previously tested mice to distinguish between potential effects of illumination as a visual cue and direct optogenetic effects on the brain. Although we did not have large enough sample sizes to characterize or quantify these effects in detail, we present them here to illustrate the types of long-term learning that can occur with visible laser cues and different reward contingencies. Because we randomly rewarded laser trials, mice could potentially learn that the laser provides a visual cue that their response is irrelevant. To address this possibility, we tested mice with the optic fiber disconnected from the implanted ferrules and instead directed at the walls of the box. In this way, illumination as a cue was clearly visible to the mouse, but had no optogenetic effect on the brain. Illumination trials were randomly interleaved on 20% of the trials. The resulting patterns of behavior support the idea that the initial effects of brain illumination (shown in Figures 1, 2) were due to optogenetic suppression of auditory cortex, but also that mice can gradually learn to associate the visual laser cue with task contingencies. For example, mouse 5982 (Figure 3A) showed an impairment on laser trials due to optogenetic suppression when illumination was delivered to the brain (left epoch of Figure 3A, “Fiber Connected”). We then disconnected the fiber but left illumination visible, after which this mouse showed almost no difference in performance between laser and control trials for approximately the first 3000 trials during this “Fiber Disconnected” control condition. Thus without direct optogenetic suppression, this mouse showed no impairment. However, over time this mouse developed a strong side bias specifically on laser trials while maintaining performance on control trials (starting around the red arrows in Figure 3A). This is consistent with the idea that after about 3000 trials, the mouse learned that the laser cue indicated a random reward. A comparison of performance on laser trials between the first 3000 trials (75.0% correct) and the following 9000 trials (68.7%) shows a significant difference using a two-tailed Fisher’s exact test (*p* = 0.0027, effect size = 1.37). Mouse 5980, which had already developed a bias during the initial laser condition, was also tested in the disconnected condition (data not shown). This mouse showed a transient reversal in side bias when the laser was disconnected, but the bias then strengthened and stabilized over time, again suggesting that the mouse learned the association between visual cue and randomly rewarded trials.

#### Normal Rewards

We randomly rewarded laser trials to avoid the possibility that mice could learn a new association between “laser + sound” stimuli and reward. To directly address whether mice could learn new associations during suppression, and confirm the necessity of randomly rewarding laser trials, we tested the impact of switching to normal rewards on laser trials, which made up 20% of the total. Fibers remained connected to the implanted ferrules for this experiment, optogenetically suppressing auditory cortex. When mice were switched from random to normal rewards, their performance on laser trials gradually improved and eventually approached or matched control trials. Importantly, this process took at least 10,000 trials. For example, mouse 5916 (Figure 3B) gradually improved performance on both control and laser trials following the switch from random rewards to normal rewards, asymptotically converging at 80% correct in both control and laser conditions after ∼15,000 trials. Three other mice showed less conclusive effects, but trended toward better performance or less bias following the switch to normally rewarded laser trials.

This gradual improvement in performance following a switch to normal rewards occurred whether or not the fiber was connected to the implanted ferrule. Indeed, switching to normal rewards could reverse the bias that had been learned during the disconnected but randomly rewarded condition. For example, mouse 5982 (Figure 3A) developed a strong bias on randomly-rewarded laser trials with the fiber disconnected, as described above. When this mouse was switched to normal rewards (with fibers still disconnected), this bias remained strong (∼90%) for 12,000 trials but then gradually diminished as the mouse presumably learned to respond correctly to normally-rewarded laser trials. Comparison of the proportion of rightward responses between the first 10,000 (88.6%) and remaining 32,000 (64.4%) trials showed a significant difference using a two-tailed Fisher’s exact test (*p* < 0.0001, effect size = 4.31). We conclude that providing normal rewards on optogenetic suppression trials can provide reinforcement that allows some mice to learn how to correctly respond, perhaps by forming a new “laser + sound” stimulus-reward association. This can occur with visual laser cues independently of optogenetic suppression of the brain. These results suggest that in experiments testing the effects of optogenetic manipulations on operant behavioral tasks, it is important to consider the reward contingency on illumination trials. Nevertheless, because the learning of these new contingencies took at least 10,000 trials, the effects of optogenetic manipulations can be measured before new associations are learned, provided one is cautious about the potential for such learning.

## Discussion

Speech evokes spatiotemporal patterns of activity in auditory cortex. Which details of these neural representations matter for discrimination of speech sounds? Here we tested the idea that early activity is more important than late activity for the discrimination of initial consonants in spoken words. By optogenetically suppressing different temporal windows of speech-evoked activity in auditory cortex of trained mice, we found that both Early and Late suppression disrupted performance equivalently. Our interpretation is that mice are impaired at recognizing either type of disrupted representation because it differs from those learned in training.

Our results show that neural activity in auditory cortex is required for the discrimination of consonants embedded in spoken words. This finding agrees with the consensus view from numerous lesion, behavioral, and electrophysiological studies. Damage to auditory cortex impairs speech discrimination in animals (Porter et al., 2011) and in humans (Miceli et al., 1978; Tallal and Newcombe, 1978; Luria, 2011). Our results extend experimental lesion results by showing that normal auditory cortical activity is required for discrimination; this distinction is important given a number of recent studies that have showed contrasting results of optogenetic suppression and lesions (Goshen et al., 2011; Kumar et al., 2013; Otchy et al., 2015; Hong et al., 2018; O’Sullivan et al., 2019). It is also well established that speech stimuli can be accurately decoded from speech-evoked activity in auditory cortex (Centanni et al., 2014), and that the accuracy of this decoding is correlated with the accuracy of trained animals across stimuli (Engineer et al., 2008; Shetake et al., 2011; Centanni et al., 2013). In particular, the initial 40 ms of the onset response is the most informative component of neural activity for consonant discrimination (Engineer et al., 2008). Our results neither support nor contradict the finding that initial activity is more important for consonant discrimination. We do not interpret the equivalent effects of early and late suppression as an indication that the two time windows contain equivalent information about consonant identity. We note that even though Late stimulus components are acoustically similar, they nevertheless contain differences that mice could use to support stimulus discrimination. From our results that Early and Late suppression produced equivalent impairment, we cannot infer which temporal components of the stimuli mice used for discrimination of intact stimuli on control trials. Rather, our findings suggest that any major disruption of auditory cortical activity is enough to disrupt performance. Even if the disruption occurs in an epoch of low informational relevance for stimulus discrimination, the altered representation is presumably different enough from the intact representations evoked by stimuli during training that the mouse does not recognize it, and shows impaired discrimination.

### Laser-Related Learning

There are experimental design tradeoffs involved in the decision of whether to randomly or normally reward laser trials. With randomly rewarded laser trials, a mouse could learn to associate any visible laser cues with the irrelevancy of its response. If a mouse learns that its response is irrelevant, it could adopt a strategy based on random guessing, or a strategy of defaulting to one side (i.e., a strong response bias), either of which could involve less effort than responding correctly to the stimulus. This learning could produce a drop in performance that has nothing to do with optogenetic suppression of the targeted brain region. Indeed, we tested for this possibility and found evidence that mice can display this kind of laser cue learning with random rewards, although it took several thousand trials. This suggests that when using randomly rewarded laser trials, it is important to measure the effects within the first few thousand trials. The appropriate number of trials likely will depend on task difficulty and the salience of any visible or neural laser cues.

Alternatively, laser trials can be normally rewarded according to the correct response for the stimulus. This avoids the possibility that mice can learn that the laser predicts that their response is irrelevant, but presents a different learning-related concern. Consistent reinforcement on laser trials could allow the mouse to learn new stimulus-reward associations for the laser + stimulus combination. For example, even if the “dad” stimulus combined with optogenetic suppression is perceived by the mouse as radically different from the intact “dad” stimulus, the mouse could use feedback from rewards to learn the correct response for a new “dad + laser” stimulus. This learning would come to obscure the effects of optogenetic suppression on discrimination. Indeed, we tested for this possibility, and found that some (but not all) mice learned to respond correctly on laser trials when they were rewarded normally. Although our sample size was small, these examples show that this type of learning is a real concern. This is why we used random rewards on laser trials in the design of our main experiments. Fortunately, this type of learning appeared to take several thousand trials. This again suggests that optogenetic effects on behavior can in principle be measured without interference from learning of new stimulus + laser combinations, provided that performance is measured within the first few thousand trials.

We used a combination of eliminating light leakage from optic fibers and continuous strobe masking to minimize the possibility that mice could learn that visible laser cues signified a randomly rewarded trial. Nevertheless, it is possible that mice could detect the laser from intracranial retinal activation, and it seems likely that they could detect optogenetic effects on the brain. Despite this possibility, our control experiments indicate that the laser effects in Figures 1, 2 were due to optogenetic suppression rather than learning that responses were irrelevant on laser trials. Detaching fibers immediately removed the impairment on laser trials, whereas laser-related learning effects took several thousand trials to develop (Figure 3).

### Future Directions

These findings confirm that normal representations of speech sounds in auditory cortex are required for behavioral discrimination. They also suggest that regardless of the amount of stimulus-relevant information in any given time window, all peri-stimulus time windows are important for behavioral discrimination. Presumably, mice were equally impaired by Early and Late suppression because neither representation matched those learned during training. Thus, we cannot infer the relative importance of different time windows of activity from our results. Nevertheless, it is clear from previous work that initial activity contains the most discriminative information about consonant identity (Engineer et al., 2008). Is there an optogenetic experimental design that could test the relative importance of different time windows of cortical speech-evoked activity? One approach would be to train animals on speech stimuli combined with optogenetic suppression, so that the representations during testing are similar to those during training. Based on our observations of laser-related learning, however, we suspect that this approach would not work. With enough training trials and appropriate reinforcement, it seems likely that mice could learn to discriminate laser–stimulus combinations as distinct representations. Another approach would be to use graded disruptions. By systematically varying the duration of the time window of optogenetic suppression, or the power of laser illumination during those windows, it might be possible to observe graded effects on discrimination performance. In essence, this might resemble a dose–response curve, and greater or lesser stimulus-relevant information in a given time window might shift this curve to left or right. One potential challenge for such an approach would be the vast number of trials required to measure such effects, and the corresponding issues of long-term laser-related learning.

## Data Availability Statement

The datasets generated for this study are available on request to the corresponding author.

## Ethics Statement

The animal study was reviewed and approved by the University of Oregon Institutional Animal Care and Use Committee.

## Author Contributions

CO’S designed the research, performed the research, analyzed the data, and wrote the manuscript. AW performed the research and analyzed the data. MW designed the research, analyzed the data, and wrote the manuscript.

## Conflict of Interest

The authors declare that the research was conducted in the absence of any commercial or financial relationships that could be construed as a potential conflict of interest.

## Footnotes

1 DeisserothLab. Predicted Irradiance Values: Model Based on Direct Measurements in Mammalian Brain Tissue. Brain Tissue Light Transmission Calculator. Available at: https://web.stanford.edu/group/dlab/cgi-bin/graph/chart.php (accessed September 1, 2019).
